# Combination Immunosuppressive Therapy Including Rituximab for Epstein-Barr Virus-Associated Hemophagocytic Lymphohistiocytosis in Adult-Onset Still's Disease

**DOI:** 10.1155/2016/8605274

**Published:** 2016-11-28

**Authors:** Eva Johanna Schäfer, Wolfram Jung, Peter Korsten

**Affiliations:** ^1^Department of Hematology and Oncology, University Medical Center Göttingen, Göttingen, Germany; ^2^Department of Nephrology and Rheumatology, University Medical Center Göttingen, Göttingen, Germany

## Abstract

Hemophagocytic lymphopcytosis (HLH) is a life-threatening condition. It can occur either as primary form with genetic defects or secondary to other conditions, such as hematological or autoimmune diseases. Certain triggering factors can predispose individuals to the development of HLH. We report the case of a 25-year-old male patient who was diagnosed with HLH in the context of adult-onset Still's disease (AOSD) during a primary infection with Epstein-Barr virus (EBV). During therapy with anakinra and dexamethasone, he was still symptomatic with high-spiking fevers, arthralgia, and sore throat. His laboratory values showed high levels of ferritin and C-reactive protein. His condition improved after the addition of rituximab and cyclosporine to his immunosuppressive regimen with prednisolone and anakinra. This combination therapy led to a sustained clinical and serological remission of his condition. While rituximab has been used successfully for HLH in the context of EBV-associated lymphoma, its use in autoimmune diseases is uncommon. We hypothesize that the development of HLH was triggered by a primary EBV infection and that rituximab led to elimination of EBV-infected B-cells, while cyclosporine ameliorated the cytokine excess. We therefore propose that this combination immunosuppressive therapy might be successfully used in HLH occurring in the context of autoimmune diseases.

## 1. Introduction

Hemophagocytic lymphohistiocytosis (HLH) is a potentially life-threatening condition characterized by excessive macrophage activity and cytokine production resulting in multiorgan failure [1, 2]. Primary HLH occurs in children with HLH-associated genetic defects or a family history of HLH, while secondary HLH develops after exposure to immunological trigger factors of underlying infections, autoimmune diseases, or malignancies [3]. In the setting of rheumatic conditions, HLH is also termed macrophage activation syndrome (MAS) and occurs most frequently in patients with systemic juvenile idiopathic arthritis (sJIA), adult-onset Still's disease (AOSD), and systemic lupus erythematosus (SLE) [4].

## 2. Case Presentation

We report the case of a 25-year-old man who had been diagnosed with AOSD five years before admission. His condition had been successfully controlled with 100 mg/d of anakinra and 5 mg/d of prednisolone. Previous attempts to reduce his immunosuppressive therapy had failed due to frequent relapses with severe arthritis and episodes of high-spiking fevers. His family history was negative for autoimmune diseases or inherited diseases.

On admission to another hospital, he presented with high fever, sore throat, and arthralgia. Laboratory tests revealed kidney and liver failure and he was transferred to the intensive care unit with suspected sepsis. His prior immunosuppressive treatment with prednisolone and anakinra was stopped and empiric antibiotic therapy was initiated. During the diagnostic process, anemia, thrombocytopenia, highly elevated ferritin levels, C-reactive protein levels, hypertriglyceridemia, hypofibrinogenemia, elevated soluble interleukin- (IL-) 2 receptor, and splenomegaly were detected. A bone marrow biopsy was performed and hemophagocytosis was demonstrated. The clinical picture was compatible with HLH/MAS according to current classification criteria, which have been developed for HLH in the context of sJIA [5]. Chemotherapy with etoposide and dexamethasone according to the HLH-94 protocol [6] was started and the patient was transferred to our hospital in slightly improved condition.

After one week, the patient deteriorated again with high fever, pancytopenia, and high levels of inflammatory markers. Physical examination on admission to our hospital was remarkable for cervical lymphadenopathy and severe tonsillopharyngitis. Computed abdominal tomography revealed hepatosplenomegaly as well as cervical and mesenteric lymphadenopathy. Chemotherapy was stopped because neutropenic fever developed and the patient appeared septic. Second-line empirical antibiotic therapy was started and dexamethasone reduced to 20 mg/d. Microbiological testing revealed a primary Epstein-Barr virus (EBV) infection with positive anti-EBV-VCA-IgM antibodies and viral load of 10^4^ copies/mL as determined by polymerase chain reaction.

**Figure 1:**
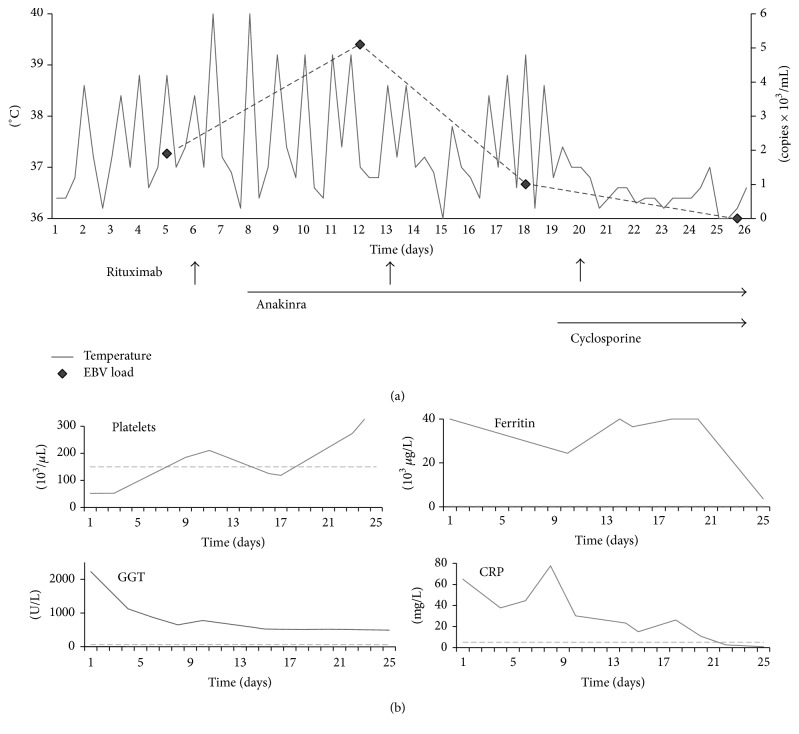
Clinical response in relation to treatment with rituximab, anakinra, and cyclosporine. (a) Fever curve and treatment overview. Body temperature was taken twice a day via an infrared ear thermometer. Rituximab was started on day 6 at a dose of 375 mg/m^2^ and repeated once a week indicated by an arrow until virus suppression was achieved after the third infusion. Anakinra was started on day 8 at a dose of 100 mg daily and cyclosporine on day 19 at a dose of 200 mg daily (blood level aiming at 100 *μ*g/L). (b) Correlating laboratory parameters over time. Dotted lines indicate the respective upper reference ranges. CRP: C-reactive protein; EBV: Epstein-Barr virus; GGT: gamma-glutamyltransferase.

We initiated treatment with rituximab once a week at a dose of 375 mg/m^2^ and achieved virus suppression after three cycles with undetectable copy numbers. Anakinra was reinstituted for AOSD and dexamethasone therapy continued at a dose of 8 mg/d. Blood count normalized and symptoms improved significantly, but quotidian fever and high levels of inflammatory values persisted. Addition of cyclosporine resulted in further clinical improvement of the patient and normalization of abnormal laboratory values. The patient course and the respective treatments are depicted in Figure 1.

## 3. Discussion

Early diagnosis of HLH in rheumatologic patients is essential to improve survival, because mortality rates ranging from 8% to 60% have been reported in the literature depending on the setting and population studied [7–11]. In general, higher mortality rates have been reported in patients with HLH secondary to underlying hematological malignancies.

The diagnosis of HLH/MAS is often delayed because of possible differential diagnoses. These include conditions that can present with similar clinical presentations and laboratory abnormalities, most importantly sepsis or systemic inflammatory response syndromes. Ferritin levels, however, are usually markedly higher in HLH patients and deemed a highly characteristic feature of HLH by most experts, which led to its recognition as mandatory criterion in the recently developed classification criteria [5]. Nevertheless, it has been argued that fever or ferritin levels can be lower in patients receiving anti-IL-1 (such as canakinumab) or anti-IL-6 therapies (such as tocilizumab) [12, 13]. Other diseases which should be excluded are infectious hepatitis, visceral leishmaniasis, brucellosis, familial hyperlipidemia, and flares of underlying conditions, notably sJIA and SLE [5, 14].

In our patient, infectious hepatitis was excluded by negative serologic testing. Visceral leishmaniasis (also termed kala-azar), which is a protozoan infection transmitted by the bite of the sand fly, can have a similar presentation. The disease is not endemic in Germany and it was very unlikely with a negative travel history of the patient to an endemic area. Brucellosis, a zoonotic disease caused by various species of* Brucella* organisms, is endemic in Germany and can present with a wide array of clinical symptoms similar to those of HLH [15]. Infection can occur by contact to domestic or wild animals including cattle, sheep, goats, or wild boars [15], making occupational exposure the most important risk factor. It has also occurred in laboratory workers exposed during routine analyses [16]. Serologic testing for brucellosis was also negative in our patient.

Recently, the so-called “HScore” has been developed for the diagnosis of reactive HLH [17]. Of note, use of different cut-off values of the HScore might be necessary in patients with underlying rheumatic conditions [18]. EBV-related HLH secondary to hematological malignancies has been successfully treated with rituximab-containing regimens, the rationale being depletion of EBV-infected B-cells [19]. In a recent case report, the use of rituximab in conjunction with chemotherapy according to the HLH-2004 protocol was unsuccessful [20]. The patient in this report also presented with a sepsis-like syndrome and was tested positive for EBV. There was, however, no underlying rheumatic condition.

In our case, on the contrary, the HLH-94 protocol was started and then terminated because of suspected sepsis. A combination immunosuppressive therapy including dexamethasone, anakinra, rituximab, and cyclosporine was necessary to achieve clinical and laboratory remission. We hypothesize that the EBV infection was the trigger for the secondary HLH in our case and led to a severe flare of the underlying AOSD. This sequence of events is probable in our view, because the clinical symptoms during the primary EBV infection (lymphadenopathy and hepatosplenomegaly) were different compared to his previous flares and rapidly disappeared upon virus suppression. By contrast, the high-spiking fevers, arthralgia, and high serological inflammatory activity persisted, which was similar to previous documented flares of his AOSD.

In conclusion, suppression of the triggering factor (EBV infection) was likely achieved with rituximab, but the severe AOSD flare and subsequent cytokine release, which occurred afterwards, required a more T-cell specific immunosuppression (i.e., cyclosporine) to be controlled. A similar approach was recently reported in a young male patient who developed secondary HLH associated with EBV with SLE as underlying autoimmune disorder [21]. We are not aware of any previous reports of rituximab-containing regimens in the treatment of EBV-related HLH in the context of AOSD and therefore propose that this combination therapy might be successfully applied in this population.

## Additional Points

(i) Secondary HLH may be triggered by primary viral infections, such as Epstein-Barr virus. (ii) EBV-associated HLH in the context of autoimmune diseases may be treated successfully using a combination immunosuppressive therapy including rituximab.
